# Sequence-Controlled Neutral-Ionic Multiblock-Like Copolymers through Switchable PIESA in a One-Pot Approach

**DOI:** 10.1021/acsmacrolett.5c00108

**Published:** 2025-08-22

**Authors:** Fabian H. Sobotta, Bas G. P. van Ravensteijn, Ilja K. Voets

**Affiliations:** † Laboratory of Self-Organizing Soft Matter, Department of Chemical Engineering and Chemistry and Institute for Complex Molecular Systems, 3169 Eindhoven University of Technology, P.O. Box 513, 5600 MB, Eindhoven, The Netherlands; ‡ Department of Pharmaceutics, Utrecht Institute for Pharmaceutical Sciences (UIPS), Science for Life, Faculty of Science, Utrecht University, P.O. Box 80082, 3508 TB, Utrecht, The Netherlands

## Abstract

Control over the composition and sequence of synthetic copolymers represents one of the most challenging targets in modern polymer science, in particular, for the labor- and time-consuming preparation of copolymers bearing ionic moieties. Though so far primarily focused on the assembly of coacervate nanostructures, we leverage polymerization-induced electrostatic self-assembly (PIESA) to achieve control over the composition and sequence of neutral-ionic copolymers and create complex chain topologies from equimolar mixtures of neutral and ionic monomers in a direct, one-pot process in aqueous solution. We are making use of the selective recruitment of charged over neutral monomers on an oppositely charged template to modulate monomer reactivities *in situ* during a controlled radical polymerization by creating segregated reaction environments. Varying the charge density of the template simply through cycling between acidic and alkaline pH drives the preferential incorporation of ionic over charge-neutral monomers by switching the template ON and OFF. Fine-tuning the length and order of the switching cycles enables the on-demand programming of specific block sequences and compositions, and even unique, alternating multiblock-like structures become accessible in a straightforward, one-pot process. Our results demonstrate a novel concept in taking advantage of the selectivity and reversibility of supramolecular compartmentalization of (charged) macromolecular building blocks to control and modulate monomer reactivities and chain topologies.

## Graphical abstract

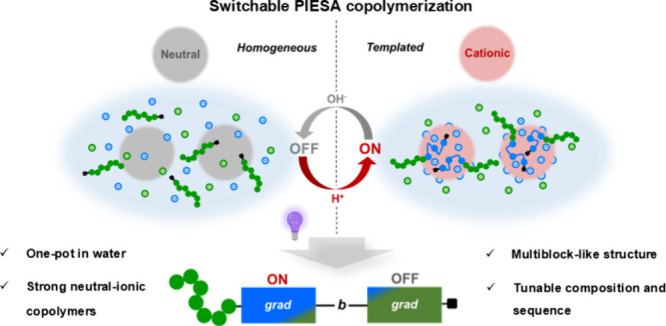

The unprecedented level of control over the monomer sequence of natural biomacromolecules facilitated by their stepwise formation is one of the key features defining their 3D structure and therefrom resulting functions. In a similar way, sequence is a key determinant of the properties of synthetic polymers at micro- and macrolength scales. For example, mechanical performance,^1,2^ bioactivity,^3,4^ and self-assembly behavior^5,6^ are sensitively dependent on this parameter. Introducing sequence regulation into synthetic macromolecules represents one of the greatest challenges of modern polymer science. Up to this date, several sophisticated strategies have been developed to create synthetic copolymers featuring uniform chain lengths and defined monomer sequences mainly exploiting iterative step- or exponential growth mechanisms, including click chemistries,^7,8^ multicomponent reactions,^9–11^ or single-unit monomer insertions.^12,13^ However, most of the reported techniques are still limited in their scale and scope of application due to being exclusively applicable to synthesize low molecular weight polymers, low flexibility in the choice of monomer, low yields, and/or time-consuming multistep procedures involving the usage of protective groups and/or catalysts.

An alternative strategy is the introduction of macromolecular templates, which, similar to nucleic acids, act as support matrices guiding monomer incorporation. Monomeric units are preorganized onto the template structure based on various mechanisms, such as covalent^14,15^ or supramolecular interactions, including hydrogen bonding,^16–18^ hydrophobic,^19,20^ and electrostatic interactions,^21–24^ as well as specific molecular recognition.^25–27^ Electrostatic interactions are of particular interest because of their generic character and straightforward implementation in existing systems.

However, up to date, the coupling of controlled polymerization techniques to electrostatic templating coined polymerization-induced electrostatically templated self-assembly (PIESA), has mainly focused on driving the assembly of nascent polyelectrolyte blocks with an oppositely charged template into coacervate nanostructures.^28–30^ Several recent studies have investigated the influence of a charged template on the copolymerization kinetics of ionic and charge-neutral monomers, as well as the possibility of obtaining complex chain topologies, including block-like^31–33^ or alternating^34^ sequences, in one step. However, all systems either rely on uncontrolled free radical polymerization or require the metered addition of a second monomer. In order to achieve the active programming of distinct, short monomer or block sequences in electrostatically driven self-assembly in a one-pot procedure, the accurate temporal and spatial coordination of both the copolymerization kinetics and the monomer/polymer–template interactions is crucial.

In a pioneering study, we have recently introduced the concept of switchable PIESA as a way to actively modulate the incorporation rates of ionic monomers *in situ* during polymerization through switching electrostatic interactions with a template ON and OFF.^35^ To achieve this, the chain extension of a macro-chain transfer agent (macro-CTA) by photoinitiated reversible-addition–fragmentation transfer (RAFT) polymerization was combined with a charge-tunable poly­(amidoamine) third-generation dendrimer (PAMAM G3) as a template to reversibly associate and confine oppositely charged monomers depending on the pH of the solution.

In this study, we envisioned leveraging the selective compartmentalization of monomer/template complexes in switchable PIESA as a tool to modulate copolymerization kinetics of binary mixtures of neutral and ionic monomers *in situ* and hence tailor copolymer compositions and sequences in one-pot (Scheme 1A).

**Scheme 1:**
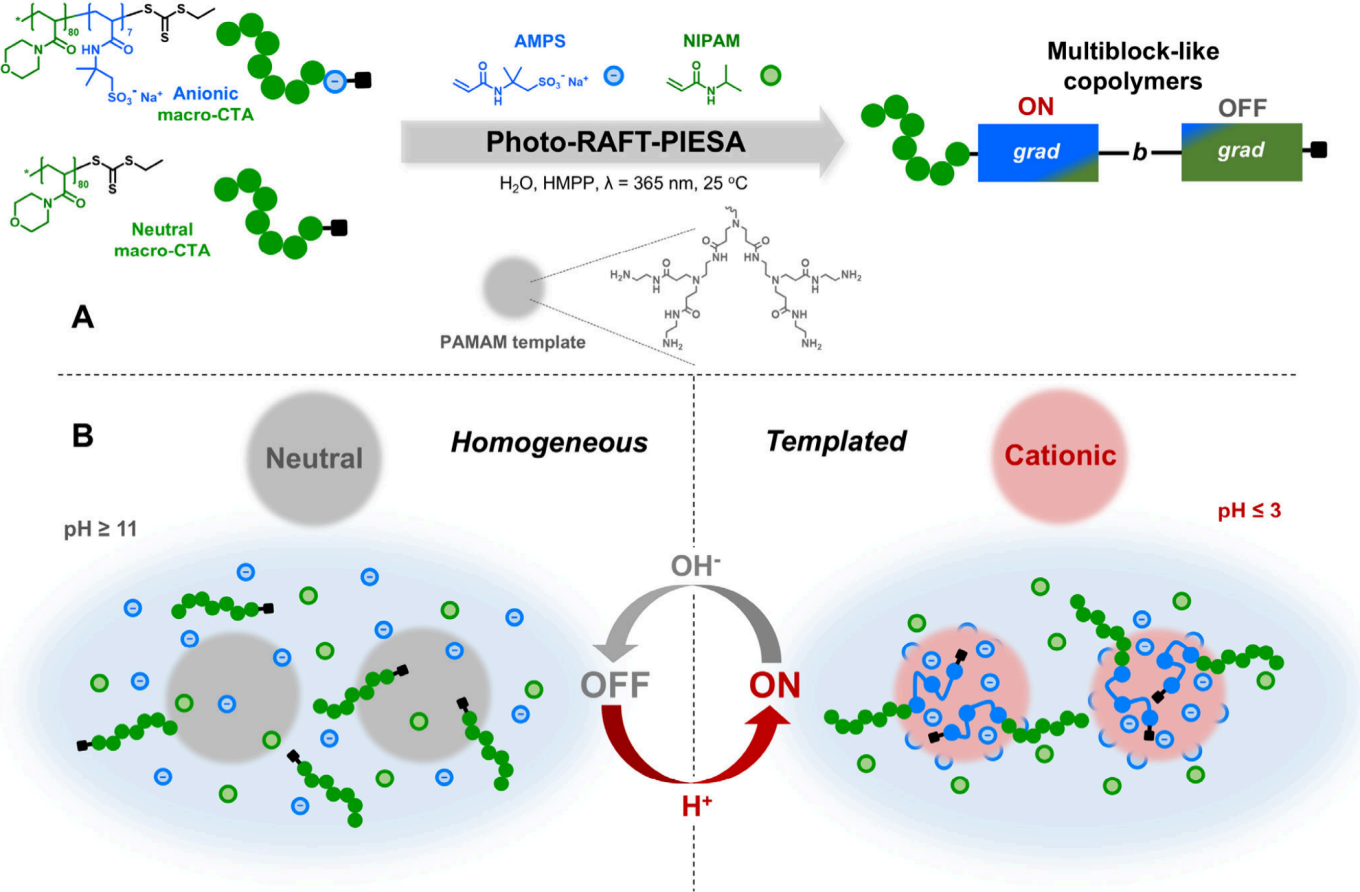
Overview of the Mechanism of Electrostatic Templating in RAFT PIESA Copolymerization

(A) Multiblock-like copolymers with controlled composition and block sequence were prepared through photoinitiated RAFT copolymerization of anionic sodium 2-acrylamido-2-methylpropanesulfonate (AMPS) and neutral monomer *N*-isopropylacrylamide (NIPAM) in the presence of neutral and anionic poly­(*N*-acryloyl morpholine) (PNAM) based macro-CTAs and a PAMAM G3 dendrimer as a charge-tunable template. The boxes represent the respective polymer segments formed either during nonactivated (OFF) or activated (ON) template conditions. The resulting copolymer composition shows a gradient distribution, which is denoted schematically in the box as *grad*. The unequal incorporation of the charged AMPS (blue) and neutral NIPAM (green) monomers along the polymer segment is visualized by a color gradient. (B) At high pH ≥ 11, the template is deprotonated (OFF) and both monomers dissolved in solution. The monomer with higher reactivity (green) is preferentially polymerized, resulting in a neutral block. At low pH ≤ 3, the template becomes protonated (ON), and the charged monomer locally upconcentrated through electrostatic interactions with the oppositely charged template, causing the selective incorporation of the charged monomer (blue) in the polymer backbone.

We hypothesize that the selective association of the charged monomer on an oppositely charged template generates a locally increased concentration of the ionic monomer compared to the neutral monomer, which remains uniformly distributed over the reaction volume (Scheme 1B). In the presence of a macro-CTA associated with the monomer/template complex, this local difference in concentration between the two monomers drives the preferential incorporation of the charged monomer over the neutral one as long as the electrostatic interaction persists.

In order to investigate the influence of a PAMAM G3 dendrimer^35^ on a PIESA copolymerization, we selected a system comprising *N*-isopropylacrylamide (NIPAM) as a neutral, water-soluble monomer and sodium 2-acrylamido-2-methylpropanesulfonate (AMPS) as a permanently charged, anionic monomer. As macro-CTAs, a neutral poly­(*N*-acryloyl morpholine)_80_ (PNAM) and a neutral PNAM_80_ chain extended with a short anionic PAMPS_7_ block carrying a trithiocarbonate end group were chosen. Using an anionic macro-CTA should associate the CTA around the complex of the monomer and cationic template from the start of the polymerization, facilitating the rapid formation of the RAFT equilibrium. The monomers were added at an equimolar ratio (1:1) to avoid any initial concentration effects on the monomer consumption. Throughout all templated copolymerizations a 2-fold excess of cationic charges on the PAMAM template to anionic monomer was maintained to ensure a sufficient number of accessible binding sites, due to the competition between anionic monomers and polymer blocks.^23^ A relatively low overall monomer concentration of 5 mM was chosen to amplify the local differences in monomer concentration between the template and the solution.^35^ Pure water was used as a solvent to facilitate the rapid switching of the pH value. Throughout all kinetic investigations, aliquots were withdrawn from the reaction mixture at certain time points and analyzed by ^1^H NMR spectroscopy to determine the conversion of both monomers (Figure S1). To reliably determine conversions of the ionic monomers by ^1^H NMR, raising the pH (≥11) was required to diminish the monomer–template interactions, as previously shown by Bos et al. and our group.^23,35^ Similarly, the determination of molecular weight distributions and separation of polymer and template signals by size-exclusion chromatography (SEC) was achieved using alkaline eluent conditions (Figure S2).

To investigate the kinetics of the untemplated copolymerization of NIPAM and AMPS, we set out to explore the photo-RAFT copolymerization kinetics of NIPAM and AMPS in the absence of a template in pure H_2_O at both acidic and alkaline pH. At acidic pH (≤3), both monomers only showed slow polymerization rates with very low conversions (<20%) even after 90 min of UV irradiation (Figure S3B,C). As expected, the low overall monomer concentration in the absence of a template significantly slows down the propagation rate.^35^ However, shifting the pH to alkaline conditions by the addition of sodium hydroxide significantly enhanced the polymerization rates and final conversions of both monomers (45% AMPS and >99% NIPAM; Figure S3E,F, Table S1). This is likely an effect of the increased ionic strength due to the addition of sodium hydroxide, as reported previously by others.^36^ It should be noted that the changes in polymerization behavior are not a consequence of cleavage of the CTA end group under alkaline conditions as evidenced by UV–vis spectroscopy measurements (Figure S4).^35^ Deriving the apparent propagation rates (*k*_p,app_) from the slopes of the pseudo-first-order rate plots (Figure S3F, Table S1) allows the quantification and direct comparison of the reactivities of NIPAM (48.2 × 10^–3^·min^–1^) and AMPS (5.8 × 10^–3^·min^–1^) at pH (≥11). The significantly faster polymerization of NIPAM compared to AMPS reflects the inherently high propensity of NIPAM to enter the growing polymer chain at the expense of AMPS, as suggested by the previously reported reactivity ratios (*r*_NIPAM_ = 2.4 ± 0.8, *r*_AMPS_ = 0.03 ± 0.02).^37^

In a second experiment, similar feed ratios and reaction conditions were maintained, but this time a macromolecular template was added to the polymerization mixture at high pH (≥11) when the template was deprotonated and therefore switched OFF. In accordance with the absence of any electrostatic interactions between the monomers and the template in the OFF state, similar polymerization rates for AMPS (9.7 × 10^–3^·min^–1^) and NIPAM (37.8 × 10^–3^·min^–1^) as well as preference for the incorporation of NIPAM over AMPS as in the absence of the template (Figure 1B) was found. The bias in reactivity toward one comonomer can be expressed as the monomer selectivity *F*, calculated based on the ratio of the respective *k*_p,app_s. Copolymerizations of AMPS with NIPAM in the absence or presence of a deprotonated, noncharged template (OFF) showed ∼ 4–8 times higher *F* for NIPAM resulting in its preferential incorporation and the formation of NIPAM enriched polymer blocks (DP_NIPAM_: 71 mol %), starting from an equimolar feed ratio (Figure 1A,E, Table S1).

**Figure 1:**
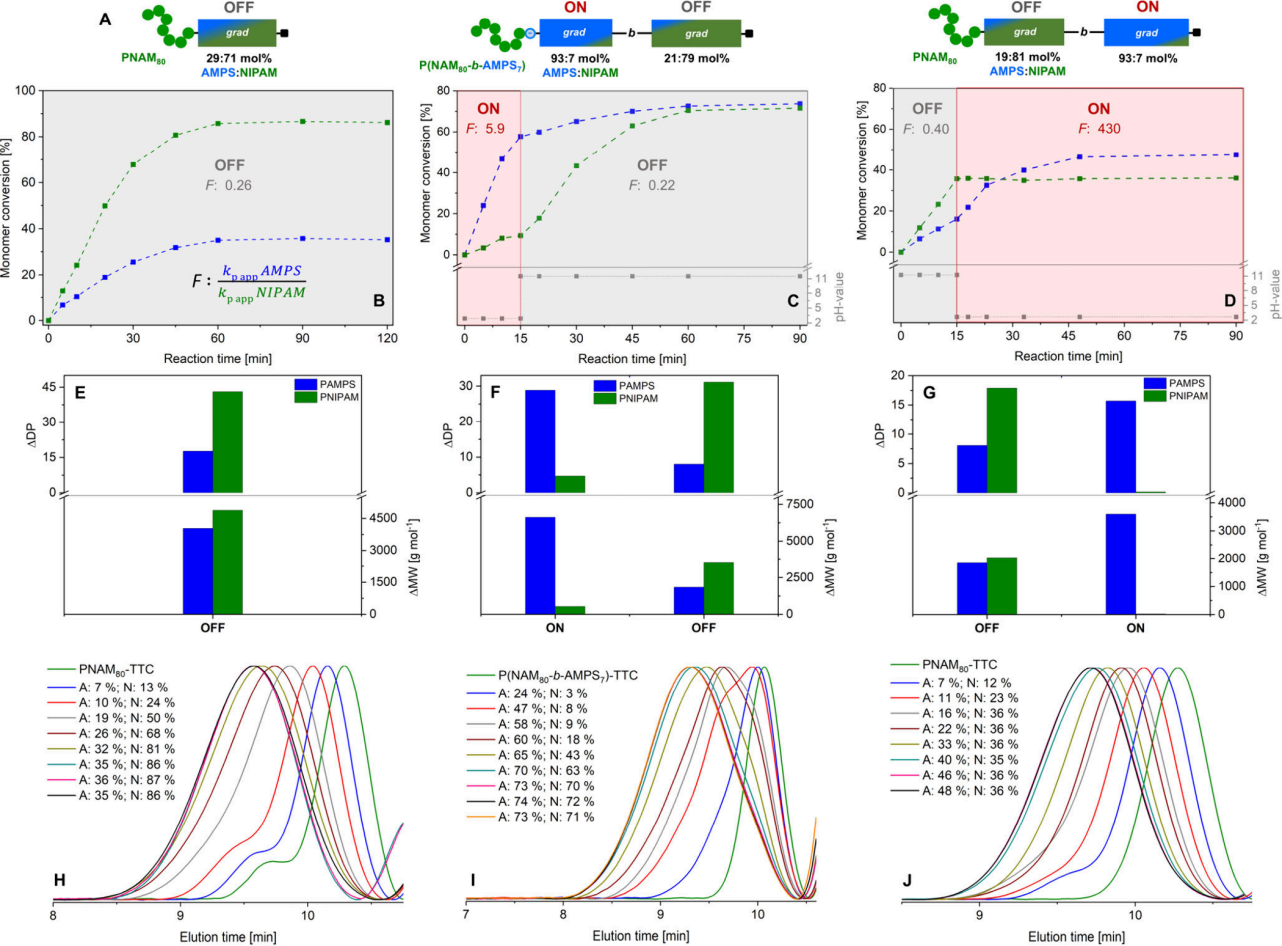
Kinetic investigation on the non- and single-switched PIESA copolymerizations. (A) Schematic depiction of the theoretical block- and sequence of the obtained block copolymers. The boxes represent the respective copolymer segments formed during the ON/OFF phases. The segments feature unequal, gradient compositions, visualized by a color gradient, as well as by the calculated average molar percentage of incorporated monomers based on their conversion. Representative conversion–time plots of copolymerizations of AMPS/NIPAM in the presence of a pH-switchable template in different protonation states. (B) The template is deprotonated during the entire polymerization (OFF, pH ≥ 11). (C) Starting from pH ≤ 3 (ON) switched to pH ≥ 11 (OFF). (D) Starting from pH ≥ 11 (OFF) switched to pH ≤ 3 (ON). The *k*_p,app_s are deducted from the slopes of the linear fit of the pseudo-first-order rate plots (Figures S5B and S7A,B) and are given in 10^–3^·min^–1^. The monomer selectivity (*F*) is defined as the ratio of *k*_p,app_ (AMPS) and *k*_p,app_ (NIPAM). The composition in the ON and OFF phase is calculated based on the changes in the individual monomer conversions between the first and last aliquot of the ON or OFF phase and represented as an average growth in the degree of polymerization (ΔDP) and molecular weight (MW) for OFF (E), an ON–OFF (F) and an OFF–ON (G) switch sequence (eqs S1 and S2). Evolution of size distributions at different monomer (A: AMPS, N: NIPAM) conversions determined by SEC for OFF (H), ON–OFF (I), and OFF–ON (J) switch sequences.

Interestingly, when performing a similar copolymerization at a low pH of ≤3 with a protonated, cationic template (ON, Figure S5A,D) the opposite was observed. The conversion of AMPS was drastically increased, while the NIPAM propagation strongly decelerated compared to the OFF state and the untemplated polymerization. In the presence of the activated template (ON), AMPS showed ∼8 times higher *k*_p,app_ (62.3 × 10^–3^·min^–1^) compared to NIPAM (8.0 × 10^–3^·min^–1^), resulting in the preferential incorporation and the formation of charged polymer blocks composed primarily (∼72 mol %) of AMPS, even starting from an equimolar feed ratio (Figure S5C,E, Table S1). The strongly disparate reactivities of AMPS and NIPAM become most apparent at the early stages of the polymerization when the conversion is still low and the concentration difference in the vicinity of the template and bulk is most pronounced. In the ON state, at low pH, the electrostatic attraction of the AMPS and the template causes a preferential incorporation of AMPS, due to the local up-concentration of charged monomer in the vicinity of the template (Figure S5D). This effect is clearly demonstrated by the differences in the *F* values for the ON (*F*: 7.82) and OFF (*F*: 0.26) states.

In both cases, i.e., in the ON and the OFF state, SEC measurements displayed stepwise, uniform shifts of the polymer size distributions to lower elution volumes up to final number-average molar weights (*M*_n_) of ∼20–23 kg mol^–1^, while maintaining low dispersities (*Đ* < 1.2), indicating the successful extension of the macro-CTAs (Figures 1H and S6C,D). For the ON state kinetics, a moderate increase in ionic strength was necessary to maintain control over the polymerization (Figure S6A vs C). The elevated ionic strength weakens the electrostatic polymer-template interactions, facilitating the diffusion of fresh monomer into the core as well as increasing the mobility of the bound polymer chain ends. Consequently, a slight reduction in polymerization rate compared to the salt-free ON kinetics was observed, due to the weakening of monomer–template interactions. In contrast, no influence of the presence of NaCl on the size distribution or polymerization rate was found for the kinetics starting in the OFF state. Additionally, the dissolution of all components at high pH enables the usage of a neutral instead of a charged macro-CTA, obtaining controlled chain growth with narrow size distributions (Figure S6B vs D). However, further in-depth studies to systematically investigate the impact of electrostatic interactions on RAFT control are necessary but beyond the scope of this work.

The above results demonstrate that the composition of the nascent polymer is readily tunable by the net charge of the template (ON/OFF) via selective recruitment of the ionic over the neutral comonomer. Polymer blocks enriched in either anionic or neutral monomers can be obtained in a single polymerization step from an equimolar mixture of comonomers.

Based on these promising findings, we aimed to investigate the possibility of switching *in situ* between the template in the ON and OFF states and thereby modulating the comonomer reactivities during the polymerization. The pH-dependent protonation state of the PAMAM template allows the reversible (de)­activation of the electrostatic monomer–template association through pH variations.^35^ We hypothesize that in the same way, the preference toward the charged or the neutral monomer should be switched *in situ* during the polymerization by cycling the solution pH between high (≥11) and low regimes (≤3; Scheme 1B). To challenge this hypothesis, we investigated the copolymerization of AMPS and NIPAM at initial equimolar concentrations. The polymerizations were started either at a high (≥11) or a low (≤3) pH and switched after certain reaction times to the opposite regime by the addition of concentrated HCl or NaOH solutions during continuous UV irradiation. The switching times were chosen such that sufficiently long PAMPS or PNIPAM blocks were able to form, without consumption of the majority of the monomers, as estimated from the monomer conversions of the continuously templated copolymerizations in the ON and OFF state.

As expected, very fast consumption of AMPS is observed when starting at a low pH in the presence of an activated template (ON–OFF, Figures 1C and S7A). Starting at a high pH with a deactivated template, NIPAM is preferably polymerized (OFF-ON, Figures 1D and S7B). The *k*_p,app_ of both comonomers before the switching point matches the values determined from the previous continuous copolymerization experiments (Table S1). As soon as the pH value was switched ON/OFF, the polymerization behavior responded instantly. This is signaled by the strong deviation in the propagation rates (Figure 1C,D), indicating that the template is able to immediately reconfigure its state through the attraction or release of monomers. However, after activation of the template from the OFF to the ON phase, a less pronounced increase in the polymerization rate of AMPS was observed compared to that of the ON-OFF sequence (Figures 1D and S7B). This might be related to the overall decrease in monomer concentration over time and the slight increase in ionic strength (∼10 mM) by the addition of HCl. Furthermore, upon activation of the template, the NIPAM propagation almost completely stopped. This peculiar behavior could be explained by the inhibited diffusion of NIPAM monomers into the highly charged polymer-template complexes, segregating them from the active polymeric radical sites. Despite the sudden change in aggregation state and monomer distribution upon switching, the evolution of the size distributions for both ON–OFF and OFF–ON sequences determined by SEC followed a continuous shift with increasing polymerization time toward lower elution times (Figures 1I,J and S7C,D). The sudden switch between two states of strongly disparate *k*_p,app,_ and *F* values results in the formation of a block-like topology comprising segregated AMPS and NIPAM-enriched blocks. Calculating the overall average number of monomers incorporated per block based on the conversion and the feed ratio, the obtained copolymer microstructure could be derived. For clarity, these are schematically depicted as boxes representing the average growth in the DP per monomer visualized by a color gradient during the alternating ON/OFF phases (Figure 1A). In the case of an ON–OFF switching sequence, an ABC-type neutral-ionic-neutral triblock-like copolymer is formed of the general structure P­(NAM_80_-*b*-AMPS_7_)-*b*-P­(AMPS_29_-*grad*-NIPAM_5_)-*b*-P­(NIPAM_31_-*grad*-AMPS_8_), whereas an OFF–ON sequence leads to a topology structurally similar to an AB-type neutral-ionic diblock-like copolymer with a compositional gradient at the interface of the neutral and anionic blocks of the formal structure PNAM_80_-*b*-P­(NIPAM_18_-*grad*-AMPS_8_)-*b*-P­(AMPS_16_-*grad*-NIPAM_0.2_). Evidently, the order of the pH switching has a major impact on the final copolymer topology.

Encouraged by these results of single-switched copolymerization, we set out to probe the experimental limits of the system and explore the integration of multiple ON/OFF switching events in a single copolymerization. The instant response of the template observed in the single-switch experiments enabled us to investigate a high number of seven switching events in total over a relatively short reaction time of ∼90 min (Figure 2B). It was expected that the progressing depletion of monomer and the increase in ionic strength due to the buildup of salt (∼10 mM NaCl per cycle) during the alternating HCl/NaOH additions would cause a slight reduction in the propagation rate of AMPS in the ON phases, as observed in the single-switch experiments. To compensate for this effect, the lengths of the ON/OFF phases were gradually extended toward the later stages of the polymerization to maintain a reasonable number of repeating units per block. On the other hand, rather short DPs of AMPS (≤10) were targeted to avoid any major interference of the charged blocks with the monomer–template complexation, as for longer polyelectrolyte chains stronger cooperative interactions can be expected.^38^

**Figure 2:**
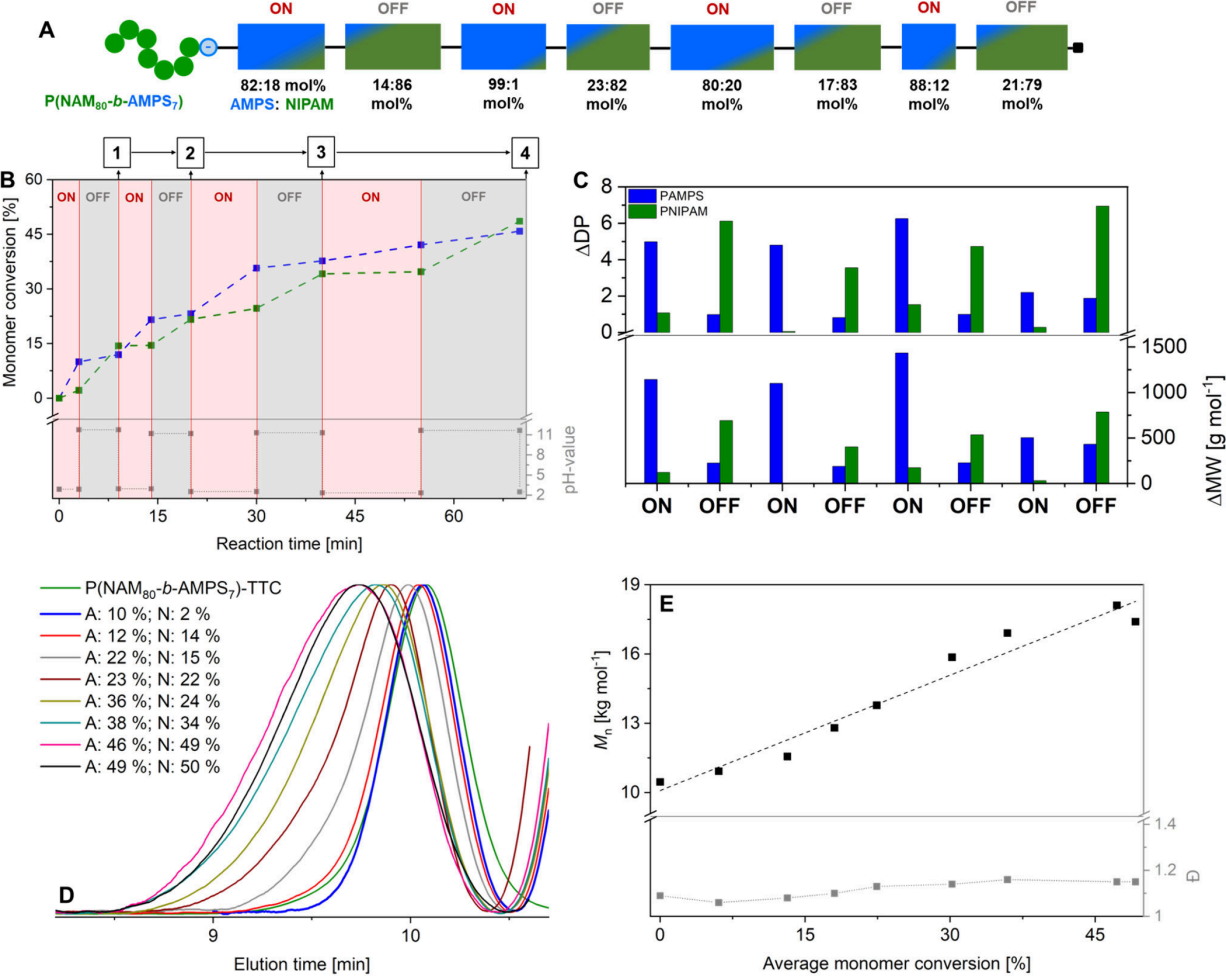
Preparation of a multiblock-like copolymer via *in situ* cyclic pH-switching. Representative conversion–time plots of copolymerizations of AMPS/NIPAM with cyclic ON/OFF switching. (A) Schematic depiction of the block- and sequence of a multiblock-like copolymer. The boxes represent the respective copolymer segments formed during alternating ON/OFF phases. The segments feature unequal, gradient compositions, visualized by a color gradient, as well as by the calculated average molar percentage of incorporated monomers based on the conversion. (B) Conversion–time plot of a copolymerization of AMPS/NIPAM with multiple pH (ON/OFF) switching events starting from equimolar, initial monomer concentrations. (C) Detailed overview of the calculated growth in DP and MW for the neutral and charged polymer comparing the individual ON/OFF phases (eqs S1 and S2). (D) Evolution of size distributions at different monomer (A: AMPS, N: NIPAM) conversions determined by SEC. (E) Evolution of *M*_n_ and *Đ* determined by SEC vs average monomer conversion. Monomer conversions are plotted as average values of AMPS and NIPAM conversions.

Figure 2B shows the conversion of both monomers to the corresponding pH values. At first sight, the anticipated ON/OFF cycling switch behavior was achieved, where AMPS polymerization primarily occurs in the ON phases and NIPAM in the OFF phases featuring an alternating stepwise growth of both polymers. The copolymerization kept on progressing over the course of four ON–OFF switching cycles, although a slowdown of the AMPS propagation becomes visible for the later ON phases. No high molecular weight byproducts were observed in SEC, which indicates a low incidence of termination reactions through chain coupling (Figure 2D). Moreover, the low maximum, average number of propagating chains per template of ∼0.14, estimated by the initial ratio of initiator to template, decreases the probability of chain coupling reaction, due to the physical segregation of radical species in different complexes, as previously reported for radical polymerizations in dispersed systems.^39^ In addition, the strong electrostatic interactions between the growing AMPS chains and the PAMAM template reduce the mobility of the polymer chains, which might further decrease the probability of chain couplings. The slowdown of AMPS polymerization at the later phases can rather be rationalized by the ongoing depletion of monomer combined with the buildup of ionic strength, as well as a gradual release of template-bound AMPS monomer by growing AMPS oligomer/polymer, due to the competition for template-binding sites, as observed for electrostatic-templated polymerizations.^23,40^ Although the general copolymerization behavior does not change, the decrease in the polymerization rate limits the experimental window in terms of achievable conversion and number of block segments. Nevertheless, the continuous, stepwise shift of the SEC size distribution to lower elution times demonstrates the successful chain extension during the switching cycles up to a final *M*_n_ of ∼18 kg mol^–1^, while maintaining low dispersities (*Đ* < 1.20, Figure 2D,E). The eight different block segments forming during the alternating ON/OFF phases exhibit disparate monomer compositions over average block lengths of ∼3 to 11 repeating units arising from the preferential incorporation of either AMPS or NIPAM (Figure 2C). The final copolymer is therefore featuring an alternating multiblock-like tapered sequence structure comprising nine segregated blocks in total, including the P­(NAM_80_-*b*-AMPS_7_) macro-CTA (Figure 2A).

Our results demonstrate the feasibility of electrostatic-templated copolymerization to reversibly modulate monomer reactivities *in situ* and the synthesis of complex polymer topologies in a one-pot procedure. The selective recruitment of charged monomers on an oppositely charged template creates a segregated reaction environment to modulate or even invert monomer reactivities *in situ* during controlled radical copolymerizations. Variation of the pH, and thus the template’s charge density, can be exploited to reversibly switch the template effect ON/OFF on demand. The system has the potential to be extended to other monomer pairs as it relies on generic electrostatic interactions responsible for modulating monomer reactivities. Exploiting the dynamic control over monomer incorporation, various multiblock-like copolymer sequences can be targeted from an equal monomer mixture, without the need for further external stimuli, specific comonomer pairs, or sequential monomer/initiator additions. Furthermore, the active programming of sequences and compositions through fine-tuning the length and order of switching cycles can be leveraged to broaden the scope of accessible sequences and access unique, intricate architectures. The accessible block lengths are comparable to previous systems based on the sequential addition of neutral or hydrophobic monomers.^41,42^ Therefore, the technique might be of particular interest for the one-pot synthesis of sequence-controlled strong ionic-neutral copolymers. These are exceptionally challenging to prepare by conventional methods, which usually involve multiple reaction and purification steps.^43^ Switchable PIESA harnesses the reversibility and specificity of supramolecular interactions combined with controlled radical polymerization techniques to gain control over the composition and sequence of forming polymer chains. The targeted positioning of charged and neutral segments within copolymer chains opens up new opportunities for the tailored design of double hydrophilic block copolymers and their assemblies.

## Supplementary Material



## Notes

Experimental procedure and additional data (PDF)
