# Supplementary material for: Sequence-Controlled Neutral-Ionic Multiblock-Like Copolymers through Switchable PIESA in a One-Pot Approach
Source: ACS Macro Lett. 2025 Aug 22;14(9):1277–83. doi: 10.1021/acsmacrolett.5c00108 (PMC12444980; doi:10.1021/acsmacrolett.5c00108)
Supplement: Supplementary file 1 [file mz5c00108_si_001.pdf]

Supporting Information

"Sequence-controlled neutral-ionic multiblock-like copolymers through switchable PIESA in a one-pot approach"

Fabian H. Sobotta,[a] Bas G. P. van Ravensteijn,*[b] Ilja K. Voets*[a]

[a]Laboratory of Self-Organizing Soft Matter, Department of Chemical Engineering and Chemistry and Institute for Complex Molecular Systems, Eindhoven University of Technology, P.O. Box 513, 5600 MB, Eindhoven, The Netherlands

[b]Department of Pharmaceutics, Utrecht Institute for Pharmaceutical Sciences (UIPS), Science for Life, Faculty of Science, Utrecht University, P. O. Box 80082, 3508 TB Utrecht, The Netherlands.

S1 Experimental part

S1.1 Materials and Methods

Hydrochloric acid (HCl, ACS reagent, 37%) was purchased from Carl Roth. Poly(amidoamine) (PAMAM) dendrimer (G3, ethylenediamine core, MeOH solution), 2-hydroxy-2-methylpropiophenone (HMPP, 97%), 4,4′-azobis(4-cyanovaleric acid) (ACVA, ≥ 98%), 1,3,5-trioxane (internal standard, ≥ 99%), sodium hydroxide (NaOH, ACS reagent, ≥ 97.0%, pellets) were purchased from Sigma Aldrich. *N*-isopropyl acrylamide (NIPAM, 98%). 2-Acrylamido-2-methylpropane sulfonic acid (AMPS) and 4-cyano-4-(((ethylthio)carbonothioyl)thio)pentanoic acid (TTC) were purchased from TCI Chemicals. The water used in all experiments was purified with a Milli-Q purification system (resistivity = 18.2 MΩ·cm at 25 °C). All other chemicals were used as received unless otherwise stated. Aqueous PAMAM stock solutions were prepared by carefully evaporating the MeOH under a stream of N_2. The given molarity is based on the total number of 62 protonable amines at pH ≤ 3 in each PAMAM molecule.

$PNAM_{80}$ was prepared *via* reversible addition-fragmentation chain-transfer (RAFT) polymerization using 4-cyano-4-(((ethylthio)carbonothioyl)thio)pentanoic acid as chain transfer agent (CTA) following a previously reported protocol.[1] Subsequent to purification by dialysis against deionized water and freeze drying, the degree of polymerization (DP) was determined by ^{1}H-NMR, and the size distribution was analyzed by size-exclusion chromatography (M_n: 7 300 g mol^{-1}, $Đ$: 1.18, poly(sodium 4-styrene sulfonate) (PSS) standard). $PNAM_{80}$-*b*-$PAMPS_{200}$ was prepared following the synthesis protocol of the charged macromolecular-CTA (mCTA) described in section S1.2.

UV irradiation was applied in a 3D-printed photoreactor chamber via an aligned UV-LED strip (λ_{max} = 365 nm, 6 V, ~ 25 W/m^2, Luxalight) at an average distance of ~ 3 cm from the reaction vial. The chamber was cooled by a connected ventilator to keep the temperature at ~ 25 ^{o}C.

^{1}H-NMR was performed at room temperature on a Bruker AC 400 MHz spectrometer in D_2O. Chemical shifts (δ) are reported in ppm relative to residual H_2O in D_2O, respectively. Samples containing 90 vol% water and 10 vol% D_2O were measured applying a water suppression pulse sequence.

Aqueous size-exclusion chromatography (SEC) measurements were performed on a Shimadzu system (LC-2050C 3D) equipped with a RID-20A refractive index detector, and a set of PSS MCX columns (5 µm, 1000 Å) with aqueous sodium hydroxide solution (0.25 M) mixed with 10 vol% dimethyl formamide as eluent at 1 mL min^{-1} at 25 ^{o}C. The system has been calibrated with sodium polystyrene sulfonate of narrow dispersity (PSS, Mainz Germany) in a molar mass range from 891 – 65 400 g mol^{-1}.

Aqueous size-exclusion chromatography (SEC) measurements were performed on a Shimadzu system (LC-2050C 3D) equipped with a RID-20A refractive index detector, and a set of PSS MCX columns (5 µm, 1000 Å) with aqueous sodium hydroxide solution (0.25 M) mixed with 10 vol% dimethyl formamide as eluent at 1 mL min^{-1} at 25 ^{o}C. The system has been calibrated with sodium polystyrene sulfonate of narrow dispersity (PSS, Mainz Germany) in a molar mass range from 891 – 65 400 g mol^{-1}.

S1.2 Synthesis of anionic mCTA P(NAM$_{80}$-b-AMPS$_7$)

For the preparation of the charged mCTA, AMPS (300 mg, 1.45 mmol, 10 eq.), PNAM$_{80}$ (1.67 g, 0.15 mmol, 1 eq.), ACVA (4.10 mg, 14.5 µmol, 0.1 eq.) and 1,3,5-trioxane (~ 3 mg) as internal standard were added to a crimp cap vial (10 ml) and dissolved in H_2O (7.27 mL). After sealing the vials with a rubber septum, the reaction mixture was deoxygenated by a stream of argon while stirring for 20 min. After taking an aliquot (~ 0.1 mL), the vial was placed in a preheated oil bath at 70 ^{o}C and allowed to stir for 17 h. After determining the monomer conversion by ^{1}H-NMR (~ 69 %), the solution was cooled to room temperature, opened to air, and purified by dialysis (MWCO: 3.5 kDa) against deionized water for three days including 4 water exchanges. Subsequent to lyophilization, the polymer was analyzed by ^{1}H-NMR and SEC.

^1H-NMR (D_2O, 400 MHz): δ = 4.01 - 3.01 (m, side chain), 2.80 - 2.32 (m, backbone PNAM), 2.23–1.99 (m, backbone PAMPS), 1.97–1.51 (m, backbone), 1.51–1.36 (m, PAMPS -CH_3), 1.36-1.10 (m, backbone).

SEC (eluent: 0.25 M $NaOH_{aq}$ + 10 vol% DMF, PSS-standard): M_n: 10 200 g mol^{-1}, M_w: 11 500 g mol^{-1}, $Đ$ = 1.13.

S1.3 (Templated) Copolymerization of AMPS and NIPAM

In a typical procedure, AMPS (216 µL of a 0.2 M stock solution in MilliQ, 43.2 µmol, 50 eq.), NIPAM (216 µL of a 0.2 M stock solution in MilliQ, 43.2 µmol, 50 eq.), P(NAM_{80}-b-$AMPS_7$)-TTC (864 µL of a 1 mM stock solution in MilliQ, 0.86 µmol, 1 eq.), freshly prepared HMPP solution (21.6 µL of a 10 mM stock solution in MilliQ, 2.16 µmol, 25 µM, 0.25 eq.), 1,3,5-trioxane (~ 2 mg) as internal standard, H_2O (6.99 mL) and PAMAM G3 (328 µL of a 0.3 M based on the total number of amines stock solution in MilliQ, 98.5 µmol, 107 eq.) were added to a crimp cap vial (10 ml). For the continuous and single-switched copolymerizations starting in the ON state, the NaCl concentration was adjusted to 20 mM by the addition NaCl solution (2 M) and the HMPP concentration increased to 50 µM (43.2 µL of a 10 mM stock solution in MilliQ, 4.32 µmol, 0.5 eq.). Prior to the polymerization, the pH of the solution was adjusted either to pH ≤ 3 for template ON or ≥ 11 for template OFF by the addition of concentrated HCl or NaOH (2 M). The vials were sealed with a rubber septum and deoxygenated by a stream of argon while stirring for 20 min. After taking an aliquot (~ 1 mL), the vial was placed in a photoreactor chamber and irradiated while stirring with UV light to initiate the polymerization. The photoreactor was kept at a constant temperature of ~ 25 °C by a connected ventilator. Throughout the polymerization an argon pressure was applied to prevent oxygen from entering the vial. The monomer conversion was monitored by taking aliquots (~ 1 mL) after certain reaction times and quenching by removing the UV light and exposing them to air.

To induce a pH switch during the copolymerization, the pH value of the aliquots was determined, and the pH of the reaction solution adjusted to ≤ 3 or ≥ 11 by the injection of 0.6 M HCl or NaOH (~ 60 µL).

For the ^1H-NMR analysis, 500 µL of the reaction solution was mixed with 60 µL D_2O and the pH adjusted to ≥ 11 by the addition of concentrated NaOH (2 M). For SEC analysis, 100 µL of the reaction solution was mixed with 100 µL of the eluent.

S1 ^{1}H-NMR analysis of copolymerization kinetics

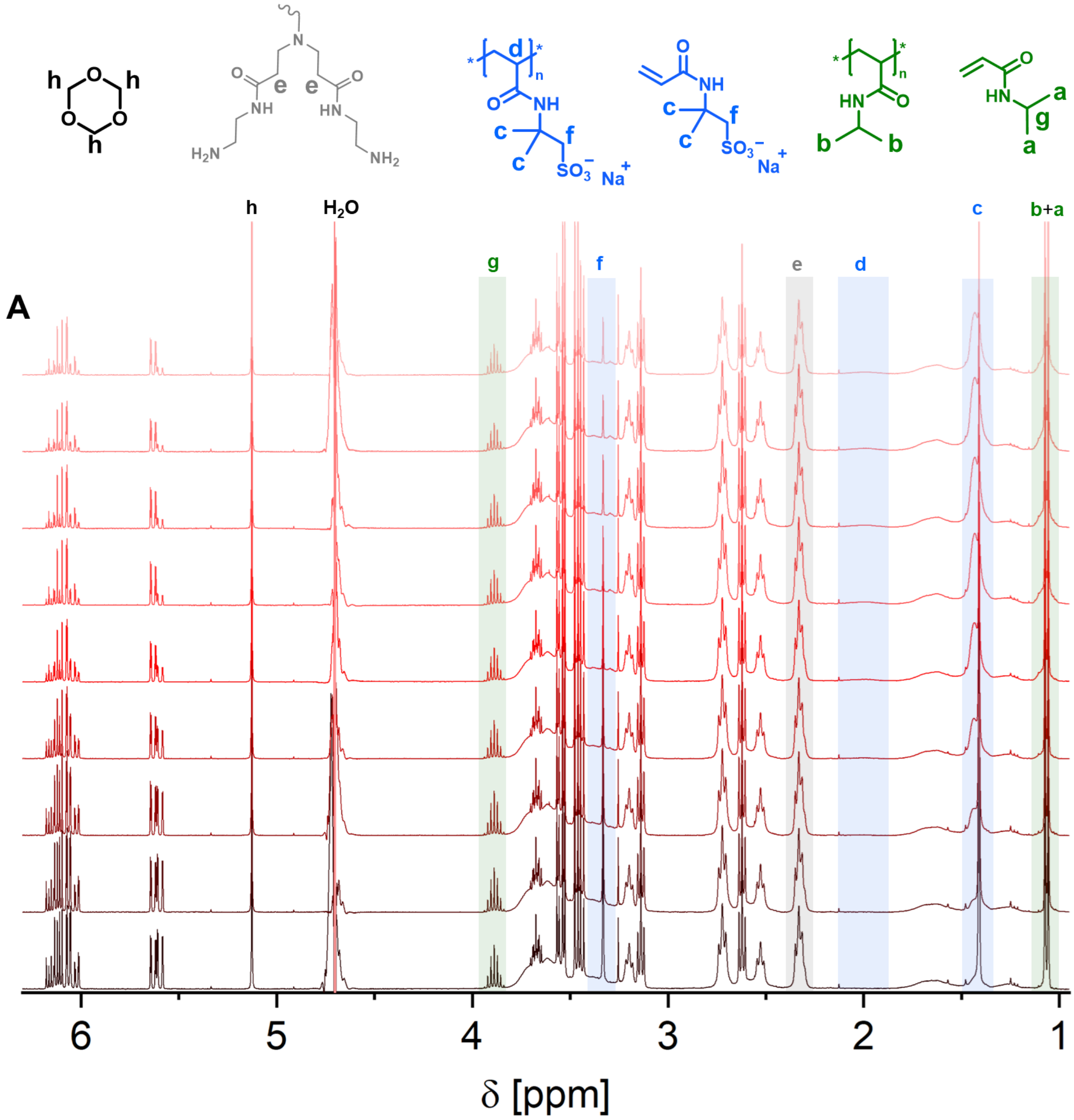

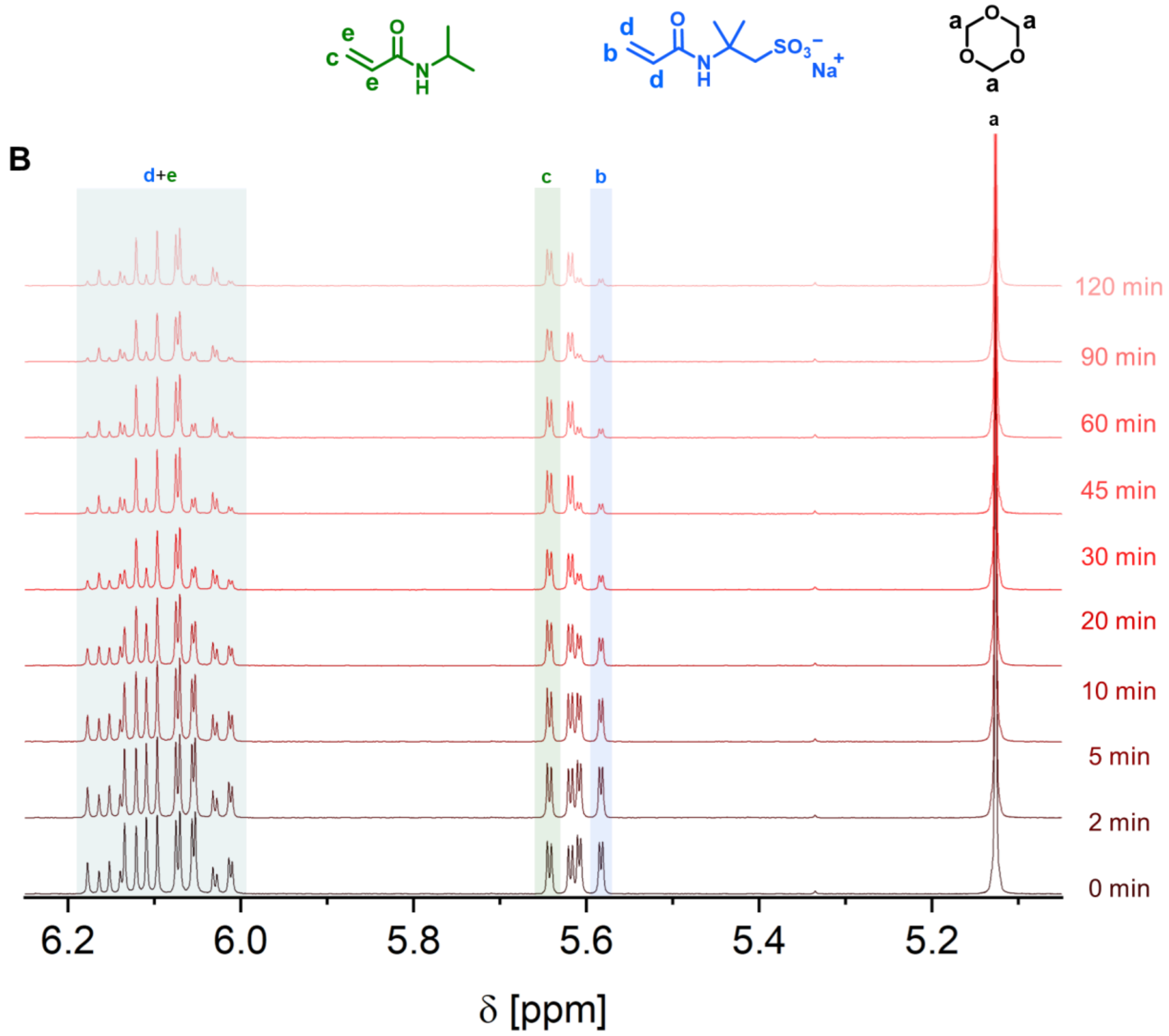

Figure S1: ^{1}H-NMR analysis of aliquots taken at different reaction times during a continuous templated copolymerization of AMPS and NIPAM at an equimolar comonomer feed in the presence of P(NAM$_{80}$-*b*-AMPS$_{7}$)-TTC as mCTA and PAMAM G3 as template. All spectra were recorded in H_2O/D_2O 9:1 mixture after the pH was increased ≥ 11 by the addition of NaOH to release unreacted monomer from the template. **A** Full spectra view highlighting the characteristic peaks for PAMAM G3 ***e*** and emerging PAMPS ***d, f*** and PNIPAM ***b*** blocks. **B** Zoom in of the vinyl proton region with characteristic peaks for 1,3,5-trioxane ***a***, AMPS ***b***, and NIPAM ***c*** monomer. Monomer conversions were calculated based on the ratio of integrated AMPS ***b*** and NIPAM ***c*** vinylic proton regions to the internal standard 1,3,5-trioxane ***a***.

S2 SEC analysis of macromolecular components

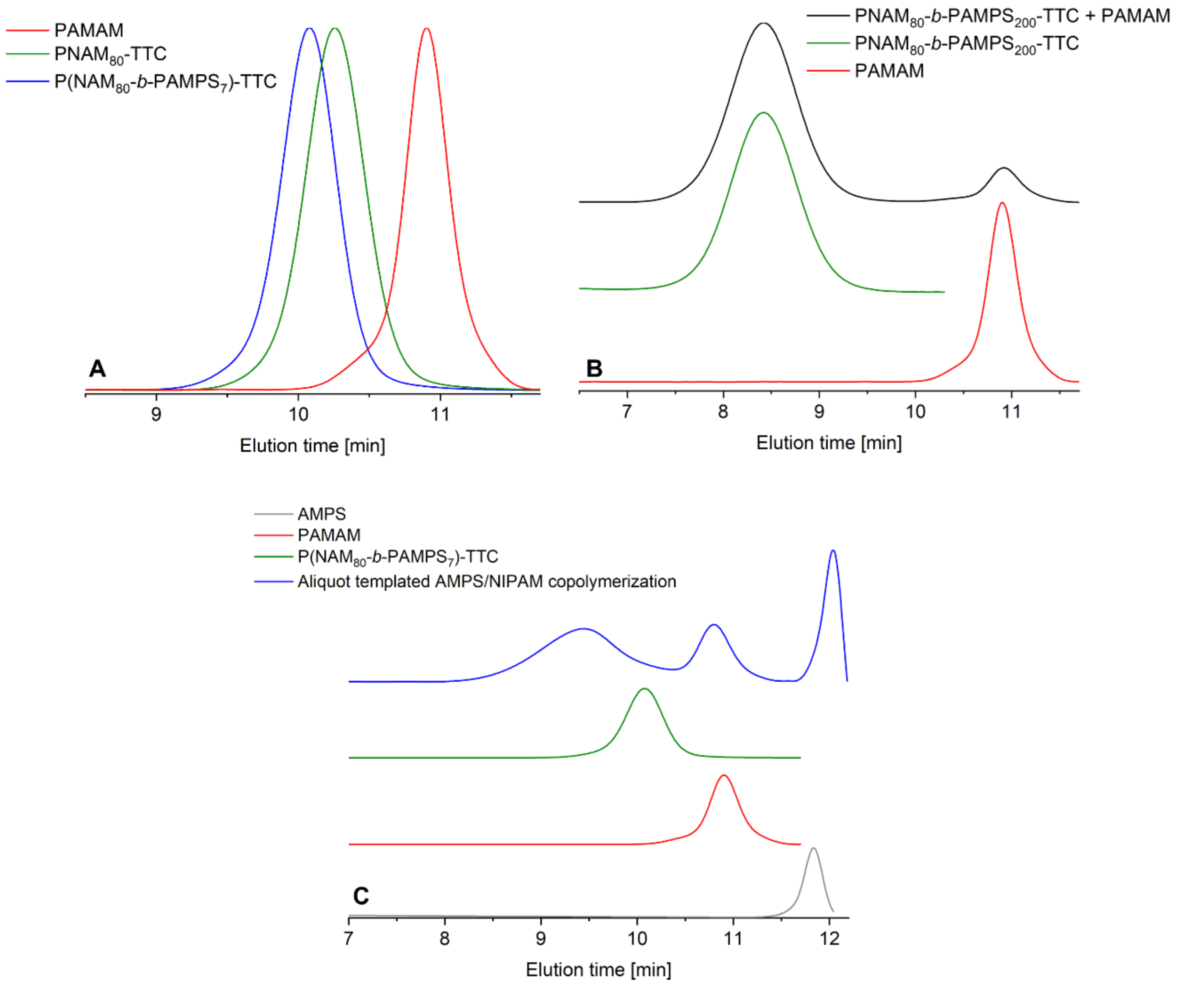

Figure S2: Overview of the SEC curves of macromolecular components of the copolymerization formulation (eluent: 0.25 M NaOH$_{aq}$ + 10 vol% DMF). **A** Size distributions of neutral and anionic macro-CTAs and pH-responsive PAMAM template used in this work. **B** Individual SEC curves of PAMAM template and PNAM$_{80}$-*b*-PAMPS$_{200}$ block copolymer compared to a mixture of both components. Uniform, narrow size distributions and baseline separation of both components in the mixture indicate the successful suppression of interactions between anionic PAMPS blocks and pH-responsive PAMAM template, as well as between PAMPS and the column material under alkaline eluent conditions. **C** Comparison of SEC curves of PAMAM template, anionic macro-CTA, and AMPS monomer with the reaction mixture of a templated AMPS/NIPAM copolymerization.

S3 Kinetic investigation of untemplated copolymerizations

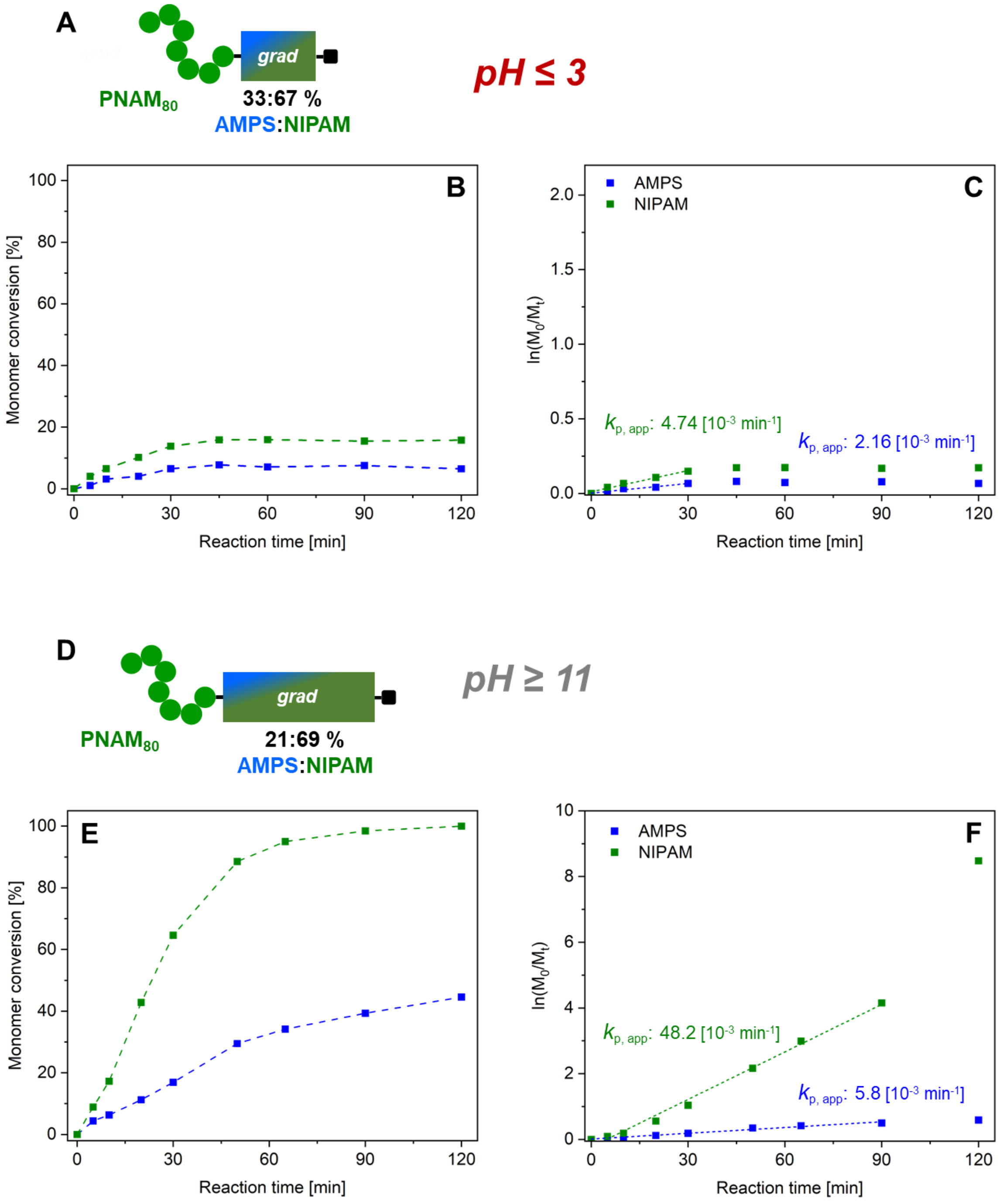

Figure S3: A, D Schematic depiction of the theoretical block composition of the obtained block copolymer in the absence of a template and NaCl at acidic and alkaline pH. The color gradient represents the calculated average molar percentage of incorporated monomers based on the conversion. Representative conversion-time **B, E** and pseudo-first-order **C, D** kinetic plots of copolymerizations of AMPS/NIPAM in the absence of a pH-switchable template and NaCl at acidic and alkaline pH. $k_{p,app}$s are deducted from the slopes of the linear fits and are given in $10^{-3}\cdot min^{-1}$ (0 mM NaCl, neutral mCTA).

S4 Investigation on hydrolytic stability of CTA chain end

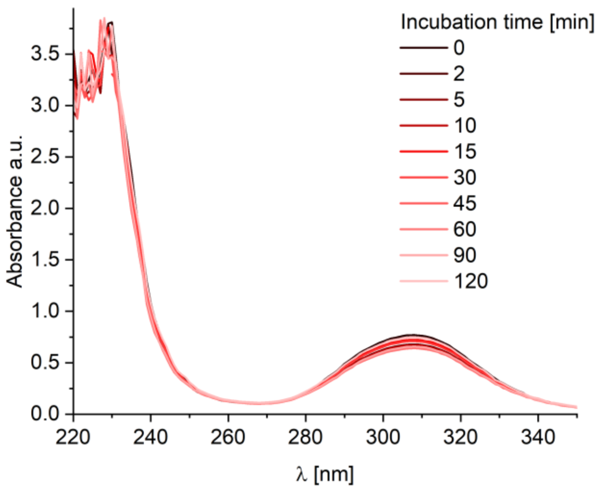

Figure S4: UV-Vis spectra of aqueous solutions of trithiocarbonate-carrying macro-CTA $PNAM_{80}$-TTC under alkaline (pH 11) conditions. The absorbance maxima at ~310 nm are characteristic of the trithiocarbonate group. Only minor reduction of CTA end groups (< 5%) was observed after an incubation time of 120 min.

S5 Kinetic investigation of continuous templated copolymerizations

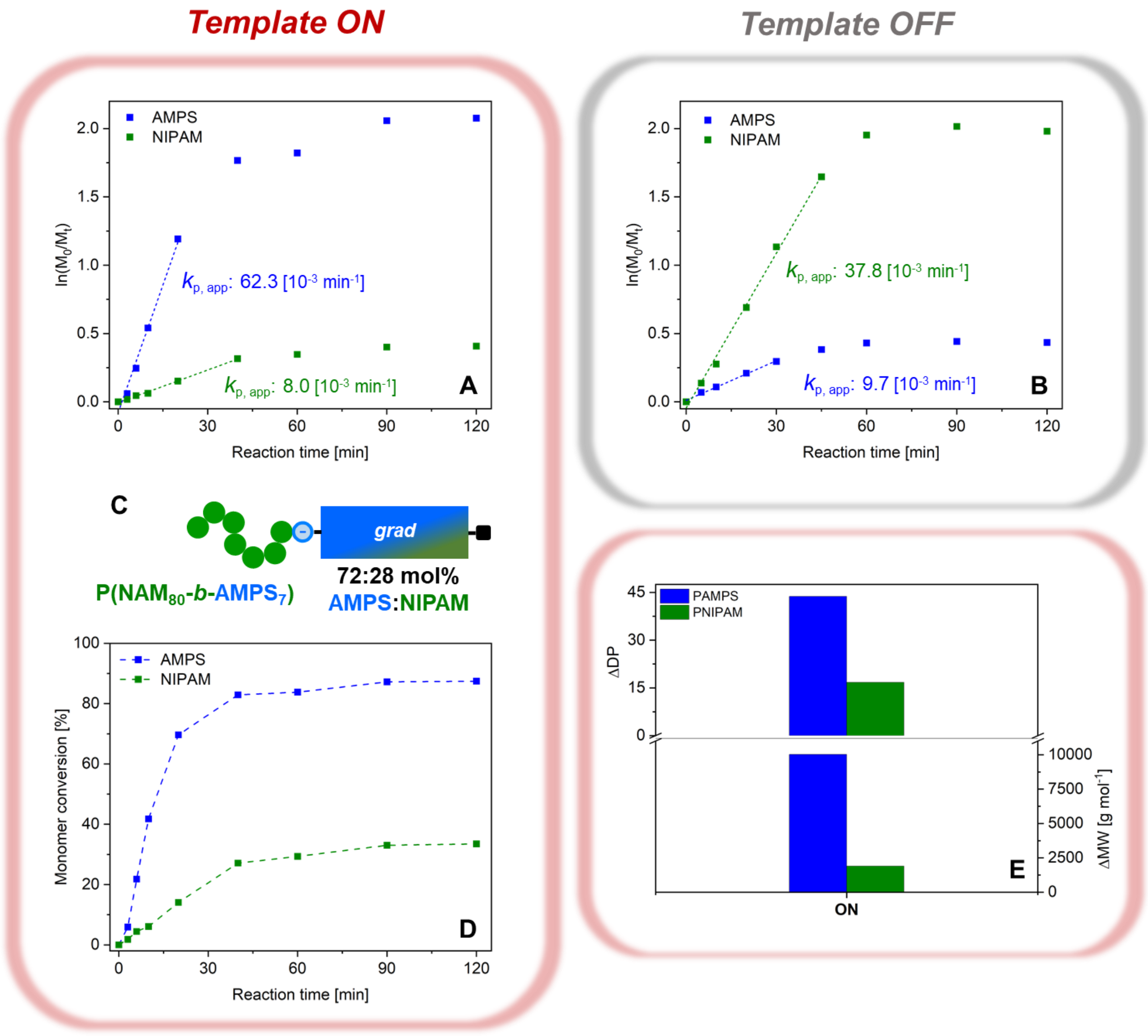

Figure S5: Representative conversion-time and pseudo-first-order rate plots of copolymerizations of AMPS/NIPAM in the presence of an activated template and NaCl (**A**, **D** ON, pH ≤ 3, 20 mM NaCl, charged mCTA) and the presence of a deactivated template with no NaCl (**B**, OFF, pH ≥ 11, 0 mM NaCl, neutral mCTA). The conversions at the individual time points were determined by ^{1}H-NMR of aliquots. The $k_{p,app}$s are deducted from the slopes of the linear fits and are given in $10^{-3}\cdot min^{-1}$. (**C**) Schematic depiction of the theoretical block composition of the obtained block copolymer in the presence of an activated template (ON, pH ≤ 3, 20 mM NaCl, charged CTA). The compositions in the ON and OFF phases were calculated based on the differences in the individual monomer conversions and represented as an average growth in the degree of polymerization (ΔDP) and molecular weight (MW) determined by ^{1}H-NMR for an activated (**E**, ON, pH ≤ 3, 20 mM NaCl, charged mCTA) template and calculated as follows:

Figure S5: Representative conversion-time and pseudo-first-order rate plots of copolymerizations of AMPS/NIPAM in the presence of an activated template (**A**-**D** ON,

pH ≤ 3, 20 mM NaCl, charged mCTA) and the presence of a deactivated template with no NaCl (**G**, OFF, pH ≥ 11, 0 mM NaCl, neutral mCTA). The conversions at the individual time points were determined by ^{1}H-NMR of aliquots. The $k_{p,app}$s are deducted from the slopes of the linear fits and are given in $10^{-3}\cdot min^{-1}$. (**E**) Schematic depiction of the theoretical block composition of the obtained block copolymer in the presence of an activated template (ON, pH ≤ 3, 20 mM NaCl, charged CTA). The compositions in the ON and OFF phases were calculated based on the differences in the individual monomer conversions and represented as an average growth in the degree of polymerization (ΔDP) and molecular weight (MW) determined by ^{1}H-NMR for an activated (**F**, ON, pH ≤ 3, 20 mM NaCl, charged mCTA) template and calculated as follows:

$$\Delta \mathrm{DP} = (Conversion\ t_n - Conversion\ t_{n-1}) \times \frac{[CTA]}{[M]} \quad (1)$$

$$MW_{Polymer} = (\Delta DP\ t_n - \Delta DP\ t_{n-1}) \times MW_{Monomer} \quad (2)$$

DP = degree of polymerization, [CTA] = concentration of chain transfer agent, [M] = monomer concentration, MW = molecular weight

S6 SEC characterization of continuous templated copolymerizations

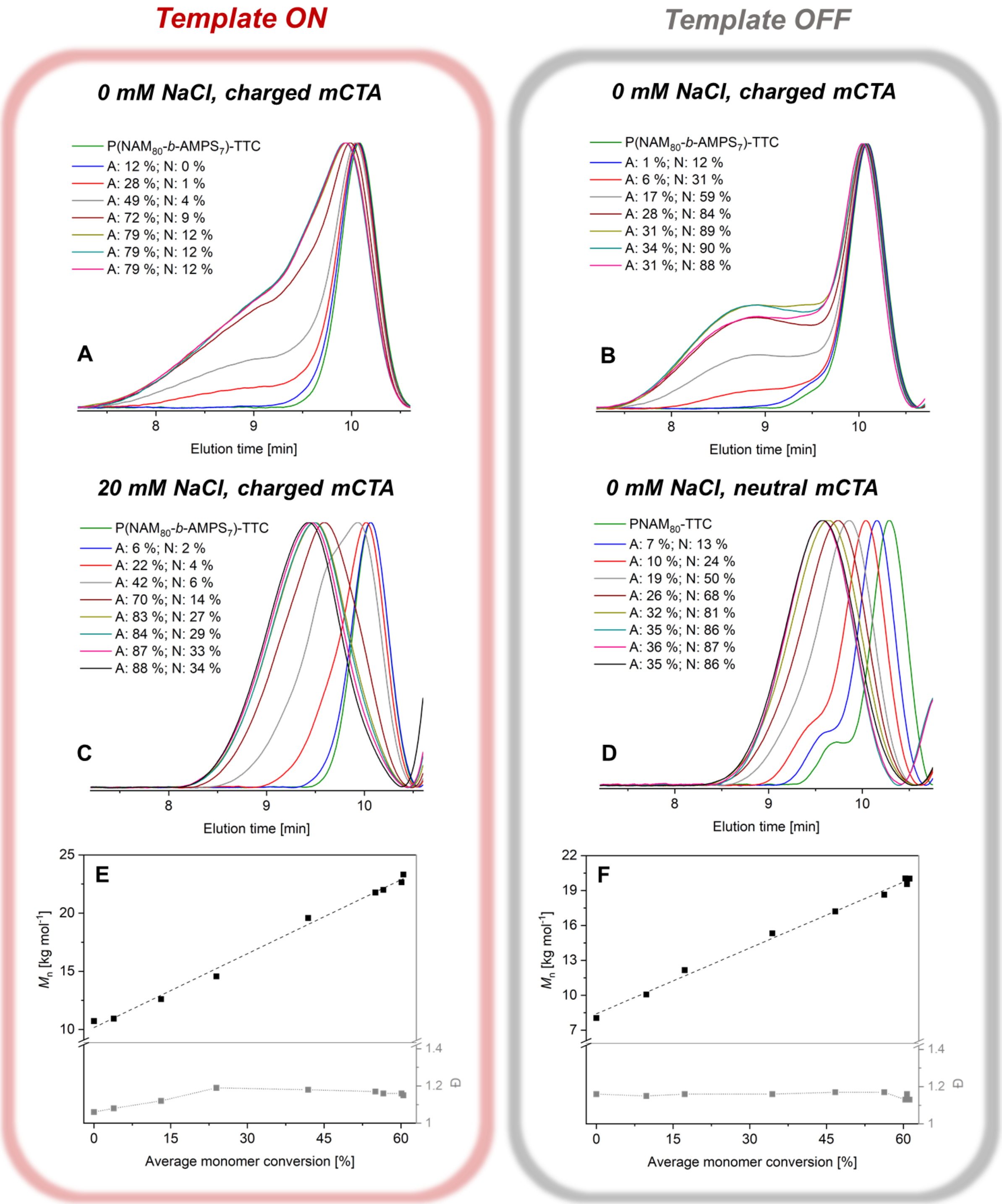

Figure S6: SEC analysis of copolymerizations of AMPS/NIPAM in the presence of an activated (**A**, **C** ON, pH ≤ 3) and deactivated template (**B**, **D** OFF, pH ≥ 11). Evolution of the size distributions at different monomer (A: AMPS, N: NIPAM) conversions in the ON state in the presence of a charged mCTA without (**A**, 0 mM NaCl, charged mCTA) and with added NaCl (**C**, 20 mM NaCl, charged mCTA). In the absence of NaCl in the ON state**,** an extreme

broadening and shoulder formation is observed, indicating the occurrence of homo- instead of block copolymerization. In the presence of NaCl, a successive shift of monomodal distributions towards lower elution times with conversion was observed, indicating a successful, stepwise chain extension of the mCTA. Evolution of the size distributions in the OFF state in the absence of added NaCl and the presence of a charged (**B**, 0 mM NaCl, charged mCTA) or neutral mCTA (**D**, 0 mM NaCl, neutral mCTA). The usage of a charged mCTA in the OFF caused extreme broadening of the SEC curve as well as the formation of a shoulder towards shorter elution times, indicating the loss of control over the polymerization. Using a neutral mCTA, a parallel shift of narrowly distributed, monomodal peaks to lower elution times was observed, indicating a controlled block extension. Correlations between molar mass and dispersity with average monomer conversion for the ON (**E**, pH ≤ 3, 20 mM NaCl, charged mCTA) and OFF (**F**, pH ≤ 11, 0 mM NaCl, neutral mCTA) states. Monomer conversions are plotted as average values of AMPS and NIPAM conversions. (RI-detection, eluent: 0.25 M $NaOH_{aq}$ + 10 vol% DMF, PSS calibration).

Table S1: Comparison of the $k_{p,app}$s, and monomer selectivities *F* of (un)templated copolymerizations. ***a*** Deducted from the slopes of the linear fits of the pseudo-first-order rate plots of the respective copolymerization kinetics. ***b*** Calculated as follows: [$k_{p,app}$ (AMPS)]/[$k_{p,app}$ (NIPAM)].

Template	**$k_{p,app}^{a}$ [10^{-3} min^{-1}] AMPS**	**$k_{p,app}^{a}$ [10^{-3} min^{-1}] NIPAM**	***F^{b}***
w/o pH 3	2.16	4.74	0.46
w/o pH 11	5.80	48.15	0.12
OFF	9.67	37.78	0.26
ON	62.29	7.97	7.82
ON-OFF	58.75 - 9.93	6.90 - 30.71	5.92 - 0.22
OFF-ON	11.59 - 29.29	27.46 - 6.44 · 10^{-5}	0.40 - 426.40

S7 Kinetic investigation of single-switched copolymerization

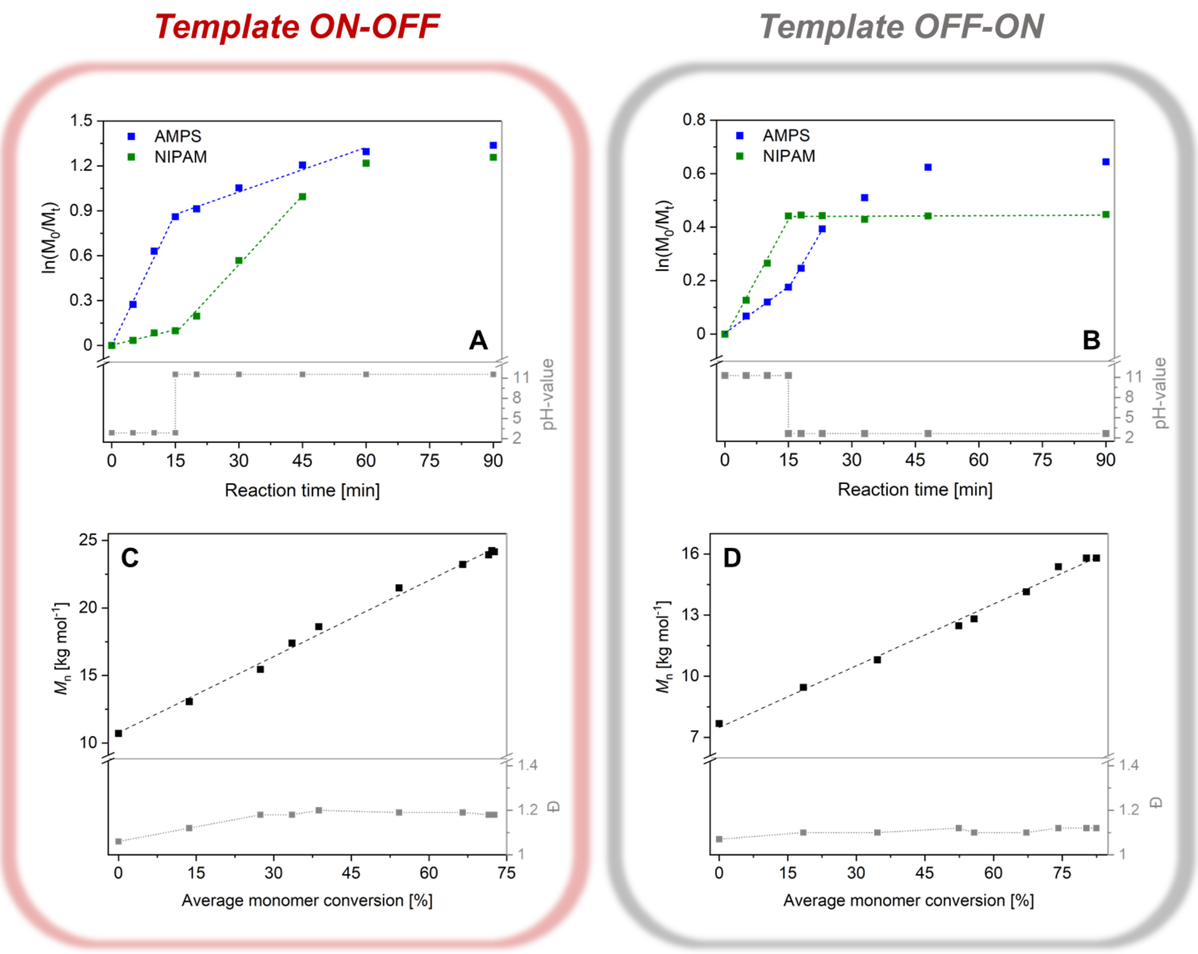

Figure S7: Representative pseudo-first-order rate plots of copolymerizations of AMPS/NIPAM with *in situ* ON/OFF switching. (**A**, 20 mM NaCl, charged mCTA) Starting from pH ≤ 3 (ON) switched to pH ≥ 11 (OFF). (**B**, 0 mM NaCl, neutral mCTA) Starting from pH ≥ 11 (OFF) switched to pH ≤ 3 (ON). The $k_{p,app}$s are deducted from the slopes of the linear fits and are given in $10^{-3}\cdot min^{-1}$. Evolutions of number-average molar mass (M_n) and dispersity with average monomer conversion for ON-OFF (**C**, 20 mM NaCl, charged mCTA) and OFF-ON (**D**, 0 mM NaCl, neutral mCTA) switching sequences. Monomer conversions are plotted as average values of AMPS and NIPAM conversions. (RI-detection, eluent: 0.25 M $NaOH_{aq}$ + 10 vol% DMF, PSS calibration)

S8 Kinetic investigation of multi-switched copolymerization

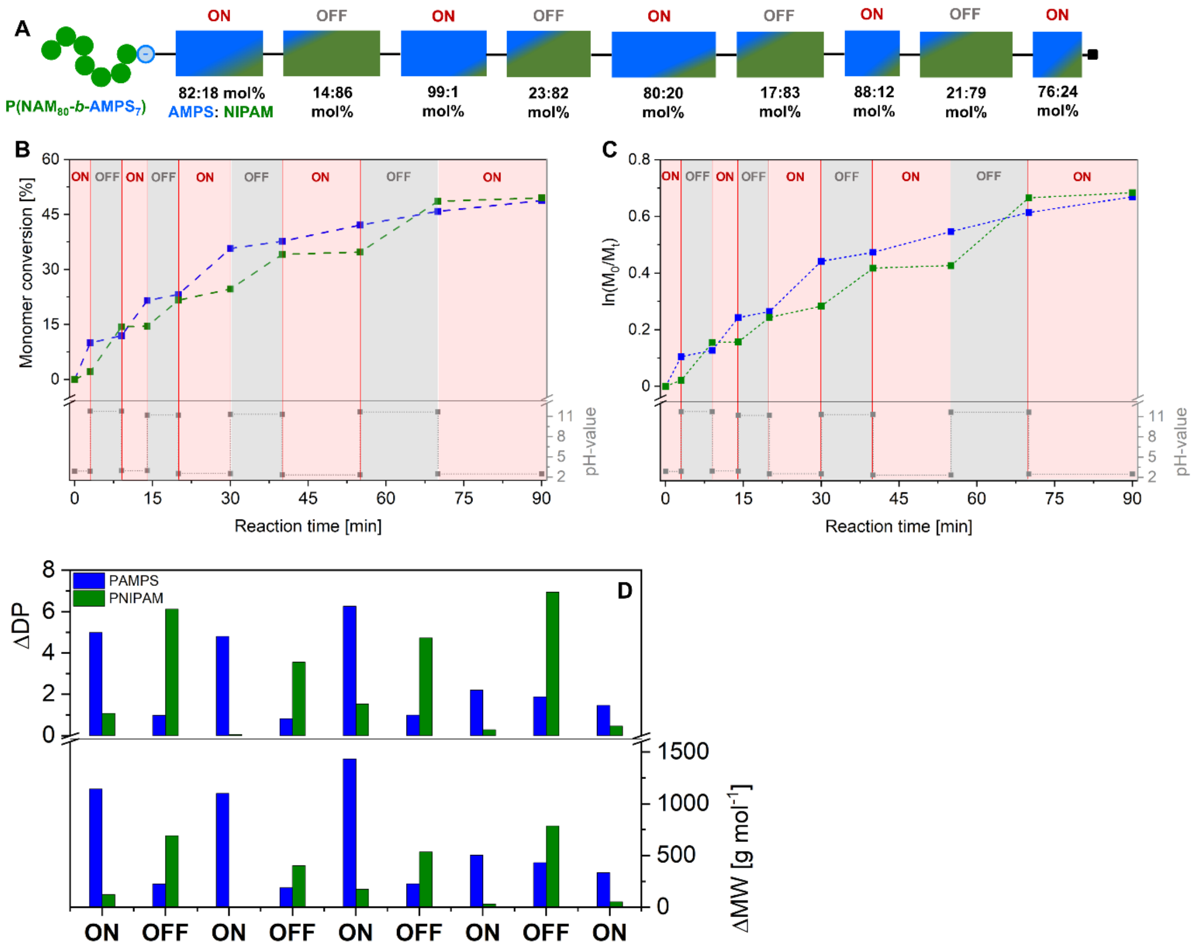

Figure S8: (**A**) Schematic depiction of the block- and sequence of a multiblock-like copolymer. The boxes represent the respective copolymer segments formed during alternating ON/OFF phases. The segments feature unequal, gradient compositions, visualized by a color gradient, as well as by the calculated average molar percentage of incorporated monomers based on the conversion. Representative conversion-time (**B**) and pseudo-first-order rate (**C**) plots of copolymerizations of AMPS/NIPAM with cyclic *in situ* ON/OFF switching (0 mM NaCl, charged mCTA). (**D**) Theoretical composition of ON/OFF polymer segments represented as the average growth in DP and MW for the neutral and charged polymer (Equation S1, 2).

References

1 Li, C.; Magana, J. R.; Sobotta, F.; Wang, J.; Cohen Stuart, M. A.; van Ravensteijn, B. G. P.; Voets, I. K. *Angew. Chem. Int. Ed.* **2022**, *61*, e202206780.
